# The Replication Function of Rabies Virus P Protein Is Regulated by a Novel Phosphorylation Site in the N-Terminal N Protein-Binding Region

**DOI:** 10.3390/v17081075

**Published:** 2025-08-01

**Authors:** Ericka Tudhope, Camilla M. Donnelly, Ashish Sethi, Cassandra David, Nicholas Williamson, Murray Stewart, Jade K. Forwood, Paul R. Gooley, Gregory W. Moseley

**Affiliations:** 1 Department of Microbiology, Biomedicine Discovery Institute, Monash University, Clayton, VIC 3800, Australia; e.tudhope@gmail.com (E.T.); cassandra.david@monash.edu (C.D.); 2 Gulbali Institute, Charles Sturt University, Wagga Wagga, NSW 2678, Australia; cdonnelly@csu.edu.au (C.M.D.); jforwood@csu.edu.au (J.K.F.); 3 Department of Biochemistry and Pharmacology, University of Melbourne, Parkville, VIC 3057, Australiaprg@unimelb.edu.au (P.R.G.); 4 Australian Nuclear Science Technology Organisation, The Australian Synchrotron, 800 Blackburn Rd, Clayton, VIC 3168, Australia; 5 The Bio21 Molecular Science and Biotechnology Institute, The University of Melbourne, Parkville, VIC 3057, Australia; nawill@unimelb.edu.au; 6 MRC Laboratory of Molecular Biology, Francis Crick Ave., Cambridge Biomedical Campus, Cambridge CB2 0QH, UK; ms@mrc-lmb.cam.ac.uk

**Keywords:** rabies virus, phosphoprotein, P protein, nucleoprotein, N protein, phosphorylation, viral replication, protein structure, mass spectrometry

## Abstract

The rabies virus (RABV) phosphoprotein (P protein) has multiple functions, including acting as the essential non-catalytic cofactor of the viral polymerase (L protein) for genome replication and transcription; the principal viral antagonist of the interferon (IFN)-mediated innate immune response; and the chaperone for the viral nucleoprotein (N protein). Although P protein is known to undergo phosphorylation by cellular kinases, the location and functions of the phosphorylation sites remains poorly defined. Here, we report the identification by mass-spectrometry (MS) of residues of P protein that are modified by phosphorylation in mammalian cells, including several novel sites. Analysis of P protein with phospho-mimetic and phospho-inhibitory mutations of three novel residues/clusters that were commonly identified by MS (Ser48, Ser183/187, Ser217/219/220) indicate that phosphorylation at each of these sites does not have a major influence on nuclear trafficking or antagonistic functions toward IFN signalling pathways. However, phosphorylation of Ser48 in the N-terminus of P protein impaired function in transcription/replication and in the formation of replication structures that contain complexes of P and N proteins, suggestive of altered interactions of these proteins. The crystal structure of P protein containing the S48E phospho-mimetic mutation indicates that Ser48 phosphorylation facilitates the binding of residues 41–52 of P protein into the RNA-binding groove of non-RNA-bound N protein (N^0^), primarily through the formation of a salt bridge with Arg434 of N protein. These data indicate that Ser48 modification regulates the cycling of P-N^0^ chaperone complexes that deliver N protein to RNA to enable transcription/replication, such that enhanced interaction due to S48E phospho-mimetic mutation reduces N protein delivery to the RNA, inhibiting subsequent transcription/replication processes. These data are, to our knowledge, the first to implicate phosphorylation of RABV P protein in conserved replication functions of the P gene.

## 1. Introduction

Rabies is a zoonotic disease with a case-fatality rate in humans of nearly 100%, and is caused by members of the lyssavirus genus (family *Rhabdoviridae*; order *Mononegavirales*, comprising the single-stranded, negative-sense RNA viruses) [1]. The prototypical species, *Lyssavirus rabies* (RABV), is the major agent of human rabies (which results in ca. 60,000 deaths annually). There is no effective treatment for rabies after the onset of symptoms, although effective prevention of disease pre- and post-exposure is possible through regimes using prophylactic vaccines and anti-RABV immunoglobulins [1].

**Figure 1:**
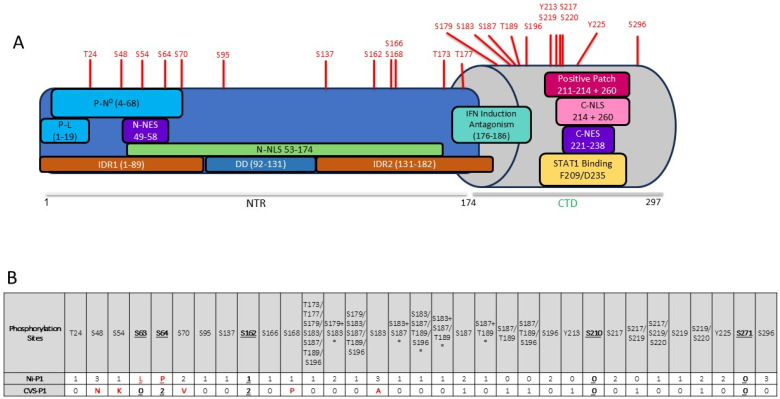
Phosphorylation sites in P1 indicated by MS analysis and/or previously predicted to be phosphorylated. (**A**) Schematic of RABV P1 protein indicating important binding/structural regions, and the phosphorylation sites identified by MS analysis (data from this study; red text above structure); P-N^0^, N^0^ binding site; P-L, L protein binding site; positive patch is involved in N-RNA-binding and C-NLS activity. (**B**) Summary of residues indicated to be phosphorylated in LC-MS/MS of Ni-P1, or previously identified as phosphorylation sites (bold and underlined); red letters indicate residues that are not conserved between Ni and CVS RABV P proteins. Summarised data from 3 independent assays with the number of assays in which phosphorylation of each residue was detected indicated; in columns where >1 site is listed, “/” indicates that the change in mass indicated that one of the sites was phosphorylated but the specific site could not be identified; “+” indicates that both sites were phosphorylated; “*” indicates phosphorylation at 2 sites.

RNA viruses have limited genome capacity due to the lack of proofreading activity of the RNA-dependent RNA polymerase (RdRp). Genomes of viruses of the order *Mononegavirales* typically encode 5 core genes (N, P, M, G, L, or their functional equivalents, and sometimes additional proteins such as a separate fusion protein) that provide structural and functional components for the viral replication cycle [2,3]. Critically, viral proteins not only mediate processes in the basic infectious cycle but also form interfaces with and regulate host processes such as innate immune signalling [4,5,6]. This is achieved in large part by multifunctional proteins such as the RABV phosphoprotein (P protein, Figure 1A). P protein functions as the essential non-catalytic cofactor of the RdRp (L protein) for genome transcription/replication [7,8], which is dependent on interaction of the P and L proteins and the nucleoprotein (N protein) in complex with viral genomic/antigenomic RNA (N-RNA, the template for transcription/replication). P protein also binds to newly synthesised N protein in its non-RNA bound form (N^0^) and acts as a chaperone to maintain N^0^ in a soluble form and deliver it to the viral genomic RNA [9].

Functions of P protein at the virus–host interface involve interactions with cellular proteins including components of the antiviral interferon (IFN) system (signal transducers and activators of transcription (STATs); Ikkε; TBK1; promyelocytic leukaemia protein, PML protein); and other cellular factors including nucleolar proteins (nucleolin, Treacle); focal adhesion kinase; and the nuclear import/export (importins, exportins) and cytoskeletal (microtubules (MTs), components of dynein) transport systems [10,11,12,13,14]. As a result, P protein is the major antagonist of IFN-dependent antiviral responses, as well as having roles in regulating other cellular processes [4,13].

A major mechanism enabling P-protein multifunctionality is its multi-modular structure comprising an intrinsically disordered N-terminal region (IDR1, residues 1–89), followed by a structured dimerization domain (DD), intrinsically disordered region 2 (IDR2, residues 131–182), and a globular C-terminal domain (P_CTD_) [15] (Figure 1A). These modules provide several discrete interaction domains/sequences, although recent data indicate that multifunctionality also derives from a higher-order spatial organisation of the domains/IDRs [9,15]. P:L protein interaction requires the N-terminal residues 1–19 of P protein [7,8], with residues 51–87 also contributing to the interface [16] (Figure 1A). The P:N-RNA interaction involves binding of a positive patch on the P-protein C-terminal domain (P_CTD_, Figure 1A) to a flexible loop of the N protein (N-loop, in which phosphorylation of the Ser389 enhances the interaction) suggesting a site for targeting by antivirals [17,18,19]. The chaperone function of P protein is mediated by its intrinsically disordered N-terminus that behaves as a molecular recognition element [20]. In the crystal structure of the P:N^0^ complex of RABV challenge virus standard 11 (CVS-11) strain, P-protein residues 4–39 form two α-helices that dock into two grooves on the N protein [21]. In addition to the crystal structure, SAXS data indicated that 50% of the N^0^:P complex population had an extended N^0^:P interface, with the P-protein residues 40–68 occupying the RNA binding groove of N protein [21].

The P_CTD_ forms a hub of interactions via several interaction surfaces/sites (Figure 1A). The N-RNA binding patch of P_CTD_ appears to contribute to importin-binding as a non-classical nuclear localization sequence (C-NLS) that is closely associated with a proposed nuclear export sequence (C-NES (Figure 1A)) [22,23]. Binding to STAT1 (a critical mediator of IFN signalling that is antagonised by P protein) was indicated to involve a hydrophobic pocket in the P_CTD_ [24], but structural analysis revealed that key contacts are made at distinct regions, including near residues Phe209 and Asp235 [25]. Other interaction sites mapped to the P_CTD_ (e.g., with PML protein, nucleolin, MTs) have not yet been defined. Interactions of the central and N-terminal parts of P protein include dynein light chain-association [26] and FAK binding [27]. The N-terminal region also contains a nuclear export signal (N-NES) [28], extended nuclear localization signal (N-NLS) (Figure 1A) and a potential third NES; these sequences provide additional functional diversity through regulated trafficking between the nucleus and cytoplasm for interactions implicated in immune evasion and nucleolar regulation [4,9,12,23,29]. P protein is also expressed as five protein isoforms due to progressive N-terminal truncation, giving additional functional heterogeneity. The P1 isoform (full-length P protein) includes residues 1–19 and the strong N-NES, and so is the cytoplasmic polymerase cofactor. Shorter isoforms P2-P5 lack L-protein binding capacity (due to deletion of residues 1–19) and, other than P2, N-NES function, so P3-P5 are able to localise to the nucleus at steady state [7,23,30].

Additional diversity, including in P-protein trafficking/subcellular localisation, arises through phosphorylation. P-protein phosphorylation has been characterised largely through biochemical approaches (e.g., radiolabelling, cell fractionation, in vitro phosphorylation, and phosphatase inhibitor treatments [22,31,32]). The data indicate that Ser/Thr phosphorylation occurs at multiple residues to generate two principal forms of P protein with altered mobility in gel electrophoresis, which are differentially present in virions and infected cells [31,32]. Analyses of *Escherichia coli*-expressed P protein (including mutants with substitutions of Ser/Thr residues) in in vitro assays using cell/tissue lysates and fractions indicated phosphorylation by several kinases at distinct regions including a non-CKII heparin-sensitive kinase [33]. Mutation at Ser63/Ser64 in the P-protein N-terminal region appears to prevent the latter phosphorylation [33]. It was also recently suggested that Ikkε may phosphorylate P protein, dependent on Ser63/Ser64, although direct detection of such phosphorylation has not been reported [34]. Other sites are reported to undergo phosphorylation by protein kinase C (PKC), inhibited by mutations of Ser162, Ser210 and Ser271 (S210/271 are in the P_CTD_) [33].

Several studies have assessed potential functions of PKC phosphorylation at Ser210 in P protein, using phosphomimetic (Ser210 to Asp or Glu) or phosphoinhibitory (Ser210 to Ala) mutations [22,33]. These indicated that Ser210 phosphorylation impairs nuclear localization mediated by the P_CTD_, dependent on exportin-1/CRM1 (the functional receptor for the defined P-protein NESs), which correlated with the effects observed following treatment of cells with a PKC agonist. Ser210 is close to the positive patch and C-NES in the P_CTD_ structure, suggesting that phosphorylation might modify competing import/export activities [22]. The S210D/E mutation also impairs MT interaction/bundling [35], which is dependent on the P_CTD_ and associated with antagonism of IFN/STAT signalling, indicating that Ser210 modification regulates several functions. Structural analysis of the P_CTD_ indicated no role for conformational change or direct perturbation of the positive patch/C-NLS by Ser210 phospho-mimetic mutations, although a charge effect may impact on interactions of this region [35]. However, only a minor affinity change was evident in N-loop binding (which is dependent on the positive patch) by S210E P_CTD_ compared with wild-type P_CTD_, suggesting that Ser210 phosphorylation does not strongly affect the interaction with N protein, such that any charge effect would appear to be relatively minor [35]. Structural analysis indicated that the major impact of S210E is in altering interactions of the sidechain of Asn226 from Glu228 of the proposed C-NES helix, with which it normally interacts, to Glu210 and freeing the Glu228 side chain to potentially participate in other interactions. Notably, Asn226 is the only residue in the P_CTD_ that is altered between the pathogenic Nishigahara (Ni) RABV strain and an attenuated, IFN-sensitive Ni-derivative strain, Ni-CE (Asn226 is substituted for His in Ni-CE), and is sufficient to confer altered pathogenesis to a recombinant virus [36]. Ni-CE mutations do not cause defective replication in the absence of IFN treatment, but result in increased sensitivity to IFN, which correlates with impaired IFN/STAT1 antagonism, nuclear localization, and MT association/bundling by P-protein isoforms, consistent with expected effects of Ser210 phosphorylation. Thus, Asn226:Ser210 interaction appears to regulate P protein-host interactions, with N226H potentially mimicking a constitutively Ser210-phosphorylated protein [11,35], linking phosphorylation to pathogenesis.

Although P protein phosphorylation generates multiple species with potential to regulate diverse functions, understanding of the phosphorylation profile remains largely limited to data from studies using in vitro phosphorylation of bacterially expressed P proteins, and phospho-mimetic/inhibitory mutations [31,32,33]. Furthermore, roles of phosphorylation at sites other than at Ser210 are unclear. Here, we used mass-spectrometry (MS) to assess phosphorylation of full-length P protein (P1) expressed in mammalian cells, identifying multiple sites, most of which are novel. Functional assessment of a phosphomimetic or inhibitory mutations to selected sites indicated a lack of substantial impact on IFN-antagonist function or nucleocytoplasmic localization. However, replication/transcription function was substantially impaired by phospho-mimetic mutation of Ser48 in the N-terminal region. Structural analysis indicated that Ser48 phosphorylation enhances interaction of P protein with N^0^, suggesting that binding/delivery of N^0^ to RNA may be suppressed by this modification. These are the first data, to our knowledge, implicating RABV P-protein phosphorylation in replication processes.

## 2. Methods

### 2.1. Plasmids

Constructs used for mammalian cell assays have been described previously [22,37] or were generated by PCR amplification of inserts and cloning into pEGFP-C1 (to express GFP-fused protein) or pmCherry-C1 (to express mCherry-fused protein) as previously described [22]. Constructs used for the minigenome, IFN induction and IFN signalling luciferase reporter gene assays have been described previously [24,38]. Proteins containing phosphomimetic and phosphoinhibitory mutations were generated by whole plasmid site-directed mutagenesis using Pfu DNA Polymerase (Promega (Madison, WI, USA) cat#M7741) according to the manufacturer’s instructions.

Plasmid to express the Ni-N^0^P (Ni RABV strain) chimaera in *E. coli* was generated by cloning of sequence encoding P protein residues 1–52 (including the N^0^-binding region 4–39), a TEV cleavage site, and N protein (residues 1–450) with a C-terminal His tag into the pET15(b) vector, using the NcoI/BamHI restriction sites. The Uniprot accession numbers for Ni-P protein and Ni-N protein are Q9IPJ8 and O55611, respectively. Plasmid to express the Ni-N^0^P-S48E chimaera was similarly generated with the P protein S48E and N protein with an S389E substitution, which is distant from the N^0^-P binding site. Plasmid to express the Ni-CE-N^0^P construct contained the Ni-CE-N sequence, wherein residues that differ from Ni-N (F273L, Y394H, F395L) are distant from the N^0^-binding site, consistent with potent replication function of Ni-CE virus expressing P protein of either Ni or Ni-CE [37,39]. The P protein sequence 1–52 is unchanged between Ni-CE and Ni RABV.

### 2.2. Cell Culture, Transfection and Treatments

HEK-293T and COS-7 cells were cultured in Dulbecco’s Modified Eagle Medium (DMEM) supplemented with 10% FCS (37 °C, 5% CO_2_). Cells were grown to approximately 80% confluency in tissue culture plates for transfection; cells for confocal laser scanning microscopy (CLSM) analysis were grown on coverslips. 293T cells were transfected using Lipofectamine 2000 (Thermo Fisher Scientific (Waltham, MA, USA)) for CLSM imaging and MS analysis, or FugeneHD (Promega (Madison, WI, USA)) for minigenome, IFN induction and IFN signalling reporter gene assays, according to the manufacturer’s instructions. COS-7 cells were transfected using FugeneHD (Promega (Madison, WI, USA)) for CLSM. To inhibit exportin 1-dependent nuclear export, cells were treated with 2.8 ng/mL Leptomycin B (LMB, New England Biolabs (Ipswich, MA, USA) cat#9676S) for 3 h prior to analysis [22].

### 2.3. Luciferase Reporter Gene Assays

For IFNα-dependent signalling assays, HEK-293T cells cultured in 24-well plates were co-transfected with 0.25 μg pISRE-luc (which expresses firefly luciferase under the control of an ISRE-containing promoter, Stratagene (La Jolla, CA, USA)), 0.04 μg pRL-TK (which expresses Renilla luciferase under the control of a constitutively active promoter, Promega (Madison, WI, USA)), and 0.25 μg pEGFP-C1 encoding WT or mutant P protein, or an equivalent amount of empty vector (EV, PUC18, Thermo Fisher (Waltham, MA, USA) cat# SD0051). 6 h later, cells were treated with or without 1000 U/mL recombinant human IFNα (PBL Interferon Source Piscataway, NJ, USA)) for 16 h before measurement of luciferase activity (below).

IFN-β induction assays were performed as previously described, with minor modifications [25,40,41]. Briefly, HEK293T cells cultured in 24-well plates were co-transfected with 0.25 μg pEGFP-C1 encoding WT or mutant P protein, or a negative control (CVS N protein), and with 0.25 μg of pGL3-IFNβ-luc (which expresses firefly luciferase under the control of the IFN-β promoter [42], a kind gift from Rongtuan Lin (McGill University, Montréal, QC, Canada)), and 0.04 μg of pRL-TK. To stimulate pGL3-IFNβ-luc, cells were co-transfected with 0.125 μg of RIG-I-FLAG plasmid [25,40,43] (a kind gift from Ashley Mansell (Hudson Institute, Clayton, VIC, Australia)); control samples were co-transfected with an equivalent amount of pUC18 (EV). Luciferase activity was measured 40 h post-transfection.

Luciferase reporter gene activity was assessed as previously described [25,38] using the dual luciferase assay system (Promega (Madison, WI, USA)) according to the manufacturer’s instructions, and a CLARIOstar plus plate reader (BMG Labtech (Ortenberg, Germany)). Firefly luciferase activity was normalised to *Renilla* luciferase activity; data was then normalised to specific control samples, as described in the figure legends.

Minigenome assays were performed as previously described [24]. Briefly, HEK-293T cells cultured in 12-well plates were transfected with 400 ng pRVDI-luc, 25 ng pRL-TK, 600 ng pCAGGS-N, 200 ng pCAGGS-L, and 100 ng pEGFP-C1 encoding WT or mutant P protein. Cells were lysed 48 h post-transfection in PLB (passive lysis buffer, Promega (Madison, WI, USA)) before analysis of firefly luciferase activity, as described [24].

### 2.4. Cell Imaging

Analysis of transfected living cells to assess transfection efficiency used an EVOS fluorescence microscope (Promega (Madison, WI, USA)). For CLSM analysis, living cells were imaged in phenol-free DMEM in a 37 °C heated chamber using a Nikon Eclipse C1 confocal laser scanning microscope (Nikon, Melville, NY, USA) with ×60 oil-immersion objective. Image acquisition used Nikon NIS-Elements software (version 4.13.01 build 916). Digitised confocal files were then analysed using FIJI software (NIH, (version 1.8.0_172(64-bit)) to measure the cytoplasmic fluorescence (Fc) and nuclear fluorescence (Fn), corrected for background fluorescence (Fb) for each cell. The Fn/c ratio was then calculated (Fn-Fb/Fc-Fb) for each cell to determine the relative nuclear accumulation of fluorescently labelled proteins, and these values were used to calculate the mean Fn/c, as previously described [12,44].

### 2.5. Mass Spectrometry

HEK-293T cells were harvested 24 h post-transfection and lysed in lysis buffer (10 mM Tris/Cl pH 7.5; 150 mM NaCl; 0.5 mM EDTA; 0.5% NP-40; PhosSTOP [Roche/Merck Cat#4906845001]; protease inhibitor cocktail [PIC; Sigma-Aldrich (St Louis, MO, USA) Cat#11697498001]) for 30 min at 4 °C. Cell debris was removed by centrifugation at 20,000 rpm for 10 min at 4 °C in a microfuge. The supernatant was then incubated with 10 µL of ChromoTek GFP-Trap Magnetic beads (cat# gtma, United Bio Research (Glenorie, NSW, Australia)) for 2 h at 4 °C on a rotary mixer. Beads were then washed three times (10 mM Tris/Cl pH 7.5; 150 mM NaCl; 0.5 mM EDTA; PhosSTOP; PIC) at 4 °C.

Bound proteins were eluted from the GFP-Trap beads using trifluoroethanol (TFE) with 10% formic acid. The samples were then incubated with trypsin at 37 °C overnight with gentle shaking. Samples were submitted to the Bio21 Mass Spectrometry and Proteomics Facility, University of Melbourne, for analysis using a ThermoFisher Q-Exactive Plus mass spectrometer linked to an Ultimate 3000 UHPLC (Waltham, MA, USA). The nanoLC system was set up for trap-elute and equipped with an ThermoFisher Acclaim Pepmap nano-trap and analytical column (Waltham, MA, USA). Peptides were separated using a 42 min gradient of 3 to 45% Acetonitrile/0.1% Formic Acid. The RAW data was analysed using software from Protein Metrics (BYONIC version v5.4.10) [45].

### 2.6. Western Analysis of Cell Lysates

Lysates of HEK-293T cells generated as described for GFP-Trap (above) were resolved by SDS-PAGE, before transfer to a nitrocellulose membrane for immunoblotting using rabbit anti-GFP (Abcam; Ab6556 (Cambridge, UK)) followed by secondary antibody (goat anti-rabbit IgG HRP conjugate antibody; Merck; Cat #AP307P (Rahway, NJ, USA)). Membranes were then imaged using Western Lightning Plus reagent (Perkin Elmer Cat# NEL103001EA (Walthsm, MS, USA)) and a Gel Doc™ XR+ Gel Documentation System (BioRad (Hercules, CA, USA)).

### 2.7. Protein Expression and Purification

RABV N^0^P constructs were used to transform *E. coli* BL21 (DE3) and expressed via IPTG induction with shaking (16 °C, 225 rpm) [46]. Pellets from cultures were resuspended in extraction buffer (50 mM Na_2_HPO_4_, 300 mM NaCl, 10 mM imidazole, pH 7.4) with one Complete-EDTA free protease inhibitor tablet (Roche, cat# 11873580001 (Basel, Switzerland)), homogenised and lysed with an Avestin EmulsiFlex C3 cell crusher (Avestin, Ottowa, Canada). The soluble proteins were recovered by centrifugation and, after filtration (0.22 µm), applied to a gravity-flow column containing 5 mL Talon metal-affinity resin beads. The column was washed with 50 mL of extraction buffer containing 50 mM Na_2_HPO_4_, 300 mM NaCl, 25 mM imidazole, pH 7.4, and protein was eluted and collected in buffer containing 50 mM Na_2_HPO_4_, 300 mM NaCl, 300 mM imidazole, pH 7.4. Eluted proteins were subjected to size exclusion chromatography on a HiLoad™ 16/60 Superdex™ 200 column (Cytiva, Marlborough, MA, USA) with 25 mM Tris-HCL, 150 mM NaCl, and 1 mM TCEP at pH 8.0. Protein was concentrated to approximately 60 mg/mL and stored at −80 °C until future use.

### 2.8. Protein Crystallisation, Data Collection, and Processing

The purified RABV N^0^P proteins were diluted to various concentrations in Tris-buffered saline (50 mM Tris, 125 mM sodium chloride (pH 8.0)) and directed into crystallisation trials using the hanging-drop vapour diffusion method. In 48-well crystal plates, 1.5 μL of protein was combined with 1.5 μL precipitant solution and equilibrated over a 300 μL reservoir. Ni-CE-N^0^P was crystallised at 30 mg/mL in a solution of 0.2 M magnesium acetate tetrahydrate, 20% polyethylene glycol 3350, pH 7.9, at 18 °C. Ni-N^0^P-S48E was crystallised at 4 mg/mL in a solution of 0.2 M lithium acetate trihydrate, 20% polyethylene glycol 3350, at 23 °C.

Crystals of Ni-CE-N^0^P were cryoprotected using precipitant condition plus 20% glycerol, and Ni-N^0^P-S48E with perfluoropolyether cryo oil (Hampton Research (Aliso Viejo, CA, USA)), and flash-cooled in liquid nitrogen before X-ray diffraction data were collected at the Australian Synchrotron on the MX2 beamline [47] using a Dectris Eiger 16M detector (Baden-Daettwil, Switzerland). Data reduction for Ni-CE-N^0^P was performed using XDS [48], and with DIALS [49] for Ni-N^0^P-S48E. The data were scaled using Aimless [50] before molecular replacement in PhaserMR (version 2.8.3) [51] using chain B of PDB 2GTT as a base model for Ni-CE-N^0^P, and the resulting model used for molecular replacement for Ni-N^0^P-S48E. Several rounds of model building and refinement were performed in COOT (version 0.9.8.93) [52] and Phenix (version 1.20.1-4487-000) [53] to complete the molecular models. Finalised structures were deposited and validated within the Protein Data Bank (Ni-CE-N^0^P, 8U0A; Ni-N^0^P-S48E, 8U0B), and interfaces were analysed by PDBsum [54].

### 2.9. Statistical Analysis

Prism version 5 software (Graphpad, San Diego, CA, USA) was used for statistical analysis to calculate *P* values using Student’s *t*-test (unpaired, two-tailed). If data sets failed the normality test, the Mann–Whitney (*t*-test) test was used.

## 3. Results and Discussion

### 3.1. P Protein Is Phosphorylated in Multiple Regions Including at Novel Sites

Although in vitro phosphorylation assays using mutants and cell lysates/fractions have indicated that P protein can be phosphorylated at several sites, data on the sites modified in intact mammalian cells is lacking. To assess this, we expressed in cells wild-type Ni or CVS P1 fused to GFP; this fusion enables visualisation of the expression and localization of P protein, including in living cells, in a form that is established as functional in replication/transcription, immune evasion, and trafficking [24,29]. We selected P proteins of CVS and Ni because these are particularly well characterised, including for effects of phosphorylation. Fluorescence microscopy analysis at 24 h post-transfection indicated efficient transfection and expression (Supplementary Figure S1A). Cells were lysed and subjected to immuno-precipitation using GFP-Trap before analysis by liquid chromatography with tandem mass spectrometry (LC-MS/MS) and assessment of P1 peptides (using the reference sequences Q9IPJ8 (Ni) and P22363 (CVS)) for post-translational modifications, using the Byonic advanced peptide and protein identification software to analyse B and Y ions [45] (Supplementary Figure S2). Each protein was analysed in three separate assays.

Samples were analysed for protein coverage and to identify phosphorylated residues (indicated by an 80 Da increase in peptide mass compared to the reference sequence). Several sites were identified as being modified but were not consistently identified in all assays. As the cells were not subjected to a specific experimental stimulus, the assays will detect basal levels of phosphorylation without activation or amplification of particular pathways/kinases, which may result in reduced detection or lack of detection at some sites. Nevertheless, we detected phosphorylation at multiple sites/regions, with several sites detected in two or three of the three independent assays performed (Figure 1B).

**Figure 2:**
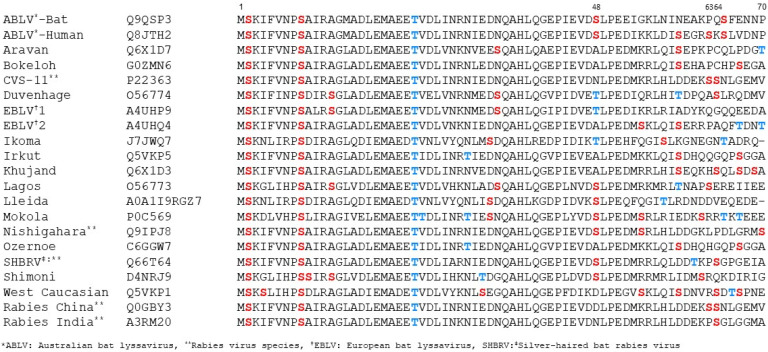
Alignment of residues 1–70 of P proteins of different lyssavirus species and RABV strains Alignment of 15 lyssavirus species (including two isolates of ABLV from bat and human, and the RABV strains silver haired bat rabies virus (SHBRV), CVS-11 and Ni, China/MRV, and India) performed using Clustal [55]. Ser (red) and Thr (blue) residues are indicated. Residues 48, and 63, 64 are indicated (see text).

Two of the phosphorylation sites detected in CVS P1, Ser64 and Ser162, correspond to potential phosphorylation sites identified previously (Ser63, Ser64, S162, Ser210, Ser271) in in vitro studies based on consensus motifs for PKC phosphorylation and deletion mapping analysis [33]. Ser162 is present in Ni and CVS P1, and modification was detected in both strains in one and two assays, respectively (Figure 1B). Ser63/Ser64 are not conserved in Ni P1 and show variable conservation across the lyssavirus genus (Figure 2). Ser63 and Ser64 are in a region that is poorly conserved and within IDR1, so is not part of a globular domain. There are multiple Ser/Thr residues proximal to Ser63/Ser64 that are variously present in P proteins of different lyssaviruses (Figure 2), including Ser48 conserved in Ni RABV, Lagos bat virus, Mokola virus, Australian bat lyssavirus (including isolates from human and bat), Shimoni bat virus, Lleida bat virus, as well as other RABV strains (including Silver-haired bat rabies virus and strains acc. no. QEU57487, AFN24101), or substituted for Thr in Duvenhage virus, European bat lyssavirus 1, Ikoma virus, or Asn in RABV strains including CVS, India; this residue was detected as a novel phosphorylation site in Ni P1 in all of our IP-LC-MS/MS assays (Figure 1B). Phosphorylation was also detected at Ser70, proximal to residues 63/64 in Ni P1, Ser70 (Figure 1B). Overall, phosphorylation in the N-terminal region appears to be conserved in Ni and CVS P1, and likely in P1 of other lyssavirus, although the specific sites may vary; the localization to a disordered region may permit flexibility in the precise localization of the modification.

Although Ser162 was reported in previous studies as a PKC phosphorylation site [33], we did not detect modification of other proposed PKC sites, Ser210 and Ser271 in either Ni or CVS P1. The lack of Ser210 phosphorylation was not unexpected, as previous analysis of P protein with an S210A phospho-inhibitory mutation (using CVS and Ni P-protein isoforms or P_CTD_ alone) show a similar phenotype to that of WT proteins in cells not treated to activate PKC, while S210D/E mutations produce a phenotype consistent with WT protein in cells following PKC agonist treatment [22,35]; these data indicate that Ser210 is largely non-phosphorylated in resting cells, consistent with our findings.

All other phosphorylation sites detected were novel, including several serines and tyrosines, indicating P protein can undergo modification by Ser/Thr- and Tyr-specific kinases. To define potential functions of modification at these sites, we selected several residues/clusters of proximal residues for further analysis based variously on parameters including location in relation to known functional sites detection in 2 or 3 separate assays, detection in Ni and CVS P1 (in cases where the residues are conserved), and close sequential proximity (Figure 1A,B): Ser48, Ser183/187, and Ser217/219/220. Other phosphorylation sites identified in this study are also likely to have functional impact on P protein and satisfy some of these criteria, and so we expect these to form the basis of future research to fully elucidate phospho-regulation of P protein. Constructs for expression of Ni P1 containing phospho-inhibitory (A) or -mimetic (E) substitutions were generated. Analysis of the phosphorylation sites of the 183, 187 and 217, 219, 220 regions identified single and double phosphorylation events but the close proximity of multiple phosphorylatable residues resulted in ambiguity of the exact site, therefore all sites in these clusters were mutated. Expression of each mutant in transfected cells was confirmed by fluorescence microscopy of living cells and western analysis of cell lysates (Supplementary Figure S1A,B).

### 3.2. Modification of Selected Phosphorylation Sites Does Not Substantially Alter P1 Nuclear Localization

Nucleocytoplasmic localization of P-protein isoforms is associated with viral replication (which is exclusively cytoplasmic), IFN antagonism, and intranuclear interactions/functions [29,56]. At steady state, P1 is almost exclusively cytoplasmic due to the N-NES, but P1 also contains NLS/NES activity (including C-NLS/C-NES, Figure 1A) and so accumulates in the nucleus when exportin-1 is inhibited by leptomycin B (LMB), indicative of active nucleocytoplasmic trafficking to produce the cytoplasmic phenotype at steady state [22]. Ser48 is immediately adjacent to the N-NES (the hydrophobic NES motif is within residues 49–58, Figure 1A), and the other selected sites, S183/187 and S217/219/220, are in the P_CTD_ which contains the C-NLS, C-NES (Figure 1A), and MT-association activity, and undergoes Ser210 phosphorylation-regulated nuclear localization [22,29,36,57].

**Figure 3:**
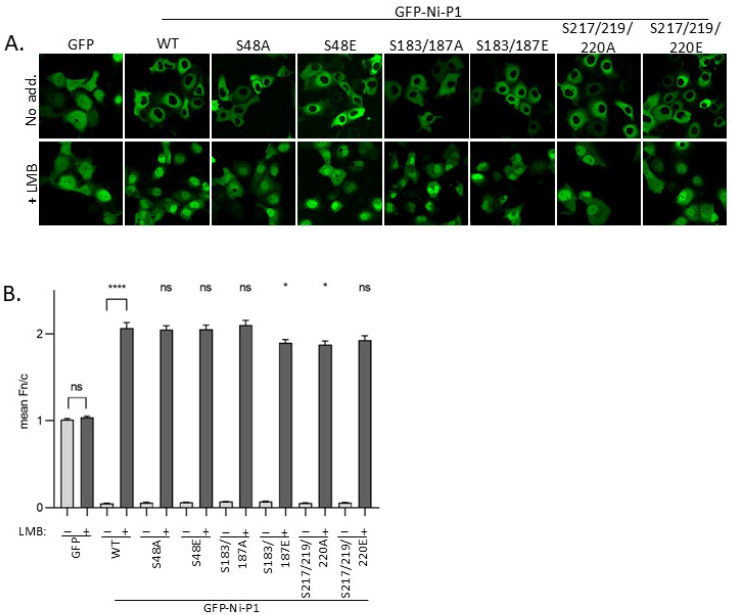
Nuclear localization of Ni P1 proteins is not substantially affected by mutations of selected phosphorylation sites. (**A**) COS-7 cells were transfected to express the indicated proteins. CLSM analysis of live cells was performed 24 h later. (**B**) CLSM images such as those shown in A were analysed to determine the ratio of fluorescence intensity in the nucleus to that in the cytoplasm, corrected for background fluorescence (mean Fn/c ± SEM, *n* ≥ 35 cells for each condition; result are from 1 assay, representative of 3 separate assays). Statistical analysis used Student’s t-test to compare the mean Fn/c for GFP or GFP-WT P1 treated without or with LMB, and the mean Fn/c for each mutant GFP-P1 protein compared to that for WT GFP-P1 protein (+ LMB). ns, not significant, * *p* < 0.02, **** *p* < 0.0001.

To assess potential effects of phosphorylation on P1 trafficking, we expressed GFP-fused WT and mutant Ni P1 proteins in Cos7 cells before treatment with or without LMB, and analysis of living cells by confocal laser scanning microscopy (CLSM), as used previously to analyse P-protein trafficking and identify important sequences [23]. GFP alone, which lacks NES/NLS activity but can diffuse through nuclear pores, appeared to have a diffuse nucleocytoplasmic localization in cells (Figure 3A). This was confirmed by quantitative image analysis, indicating a ratio of nuclear to cytoplasmic fluorescence (Fn/c) of ca. 1 (Figure 3B), which was not significantly affected by treatment of cells with LMB. All P1 proteins were strongly excluded from the nucleus (Fn/c < 0.1), which we confirmed to be dependent on exportin-1 based on a significant increase in nuclear accumulation following LMB treatment (*p* < 0.0001, Figure 3B). Thus, N-NES function is retained in the mutated proteins, as is NLS activity (indicated by an Fn/c > 1.8 for all P1 proteins following LMB treatment). However, a minor but significant (*p* = 0.02) reduction in nuclear localization was observed for P1 S183/187E, but not for P1 S183/187A, compared with WT P1 in LMB-treated cells (Figure 3B). This suggests that a negative charge at these positions moderately impacts on nuclear localization. Substitution of Ser217/219/220 for either Ala or Glu produced a similar minor reduction in nuclear localization compared with WT P1, suggesting that changes to these positions can impact on localization independently of a negative charge. Together, these data suggest that the phosphorylation sites assessed do not substantially affect P1 nuclear trafficking, although the minor impact of substitutions at S183/187 in the P_CTD_ may suggest some phosphoregulatory effect on nuclear import/accumulation. One mechanism may involve the addition of negative charge near to the positive patch/C-NLS resulting in reduced importin binding. Since mutations in the P_CTD_ can cause off-target structural effects/destabilisation [25], it is also possible that the effects observed are non-specific (particularly for S217/219/220 A and E mutations), as even small structural changes in P1 could potentially hinder interaction with nuclear import receptors. Elucidation of the nature of these effects will require structural/biophysical analysis. Due to the lack of detectable impact and/or very minor effects observed, and lack of clear impact in non-LMB-treated cells, we focussed on determining other potential functional outcomes.

### 3.3. Modification of Selected Phosphorylation Sites Does Not Significantly Impact on IFN Antagonist Functions of P1

P protein is the principal IFN antagonist of RABV, inhibiting both IFN induction and subsequent STAT1-dependent IFN signalling [6,38,57,58]. The mechanisms of antagonism of IFN induction are only partially understood, but appear to require residues 176–186 of P protein (although this region was identified by deletion analysis, and effects of deletion appear likely to include structural impact) [59]; antagonism of IFN-induction also appears to be due to inhibition of phosphorylation of IRF3/7, which is suggested to involve interactions of P protein with the kinases TBK1 and/or IKKε [59,60,61,62]. Antagonism of IFN signalling is dependent on direct binding of the P_CTD_ to STAT1, which inhibits IFN-activated nuclear accumulation and DNA interaction/IFN stimulated gene (ISG) activation by STAT1 complexes [24,25,63]. Multiple phosphorylation sites are proximal to/within residues 176–186 and the STAT1-binding P_CTD_ (Figure 1A). Ser48 is proximal to the N-NES that mediates nuclear export of P-STAT1 complexes, which is required to prevent STAT1 nuclear accumulation [37,63]. While the N-NES appears to retain strong activity in the Ser48 mutant P1 protein (above), Ser48 mutation/phosphorylation may impact on the capacity to export STAT1 complexes, or on other relevant inter- or intramolecular interactions of P1.

**Figure 4:**
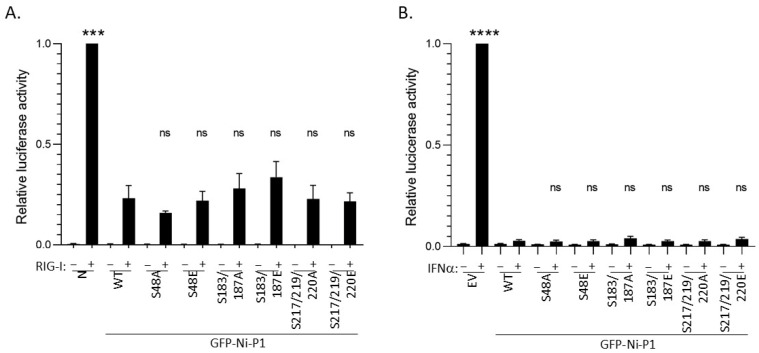
P1 proteins mutated at selected novel phosphorylation sites retain IFN antagonist functions. 293T cells were transfected to express the indicated proteins together with pRL-TK, from which Renilla luciferase is expressed constitutively, and (**A**) pGL3-IFNβ, in which firefly luciferase expression is controlled by the IFNβ promoter, with empty vector (EV, −) or plasmid to express RIG-I (+), or (**B**) pISRE-luc, in which firefly luciferase expression is controlled by an ISRE-containing type-I-IFN-dependent promoter. For (**A**), cells were lysed 40 h post-transfection for analysis by a dual luciferase assay to determine luciferase activity (ratio of firefly/Renilla luminescence). Technical triplicate values in each of 3 separate assays were normalised to values for N protein (N) + RIG-I in the same assay, and the average for the 3 separate assays was calculated (mean relative luciferase activity ± S.E.M, *n* = 3 for RIG-I-activated samples). For (**B**) cells were treated at 24 h post-transfection without or with IFNα, and lysed 16 h later for a dual luciferase assay; luciferase activity (firefly/Renilla) for technical triplicate values in each of 3 separate assays was normalised to that for EV + IFNα in the same assay, and the average value from the 3 assays was determined (mean ± S.E.M, *n* = 3 for IFN-treated samples). Statistical analysis used Student’s t-test to compare the relative luciferase activity in RIG-I or IFNα-activated cells for samples transfected to express N protein/EV controls or mutant P1 proteins with that for cells transfected to express WT P1. *** *p* = 0.0003; **** *p* < 0.0001; ns, not significant.

To examine effects of P1 mutations on IFN antagonism, we used dual luciferase reporter assays that measure (1) activation of the IFNβ promoter by the pattern recognition receptor RIG-I (which is known to recognise RABV, and to activate downstream signalling via TBK1/IKKε/IRF3 that is inhibited by P protein [24,64]) (Figure 4A), or (2) activation of a type I IFN/STAT1/2-dependent IFN-stimulated response element (ISRE)-containing promoter by treatment of cells with IFNα (Figure 4Β) [25,40].

As expected, in cells transfected with negative controls (empty vector (EV, pUC18) or vector expressing RABV N protein, which lacks discrete IFN-antagonist function [65]), RIG-I transfection or IFNα treatment caused strong induction of luciferase controlled by the IFNβ promoter or ISRE-containing promoter, respectively (Figure 4A,B). In both cases, the induced luciferase expression was inhibited by WT P1 protein (as expected [38,61]), and all mutants produced comparable inhibition (Figure 4), with no significant difference in relative luciferase activity observed for any mutant compared with WT P1. Thus, the data do not indicate major roles for phosphorylation at these sites in regulating IFN antagonist function of P1. This is consistent with a lack of substantial effects of the N-terminal mutations on P1 nuclear export, and with the fact that none of the residues correspond to residues indicated to form part of the defined P-STAT1-binding sites [25]. Furthermore, the data suggest a lack of significant impact of any of the mutations on P_CTD_ structure (as STAT1-binding appears to be sensitive to destabilisation of the P_CTD_ [25]), which further agrees with the observation of little or no impact on nuclear localization activity (Figure 4). While the precise sites/sequences and interactions responsible for P1-dependent antagonism of IFN induction are only partially understood, our data suggest that phosphorylation proximal to or within sites currently implicated does not have major regulatory roles in these processes.

### 3.4. S48E Impacts on P Protein Function in Transcription/Replication

**Figure 5:**
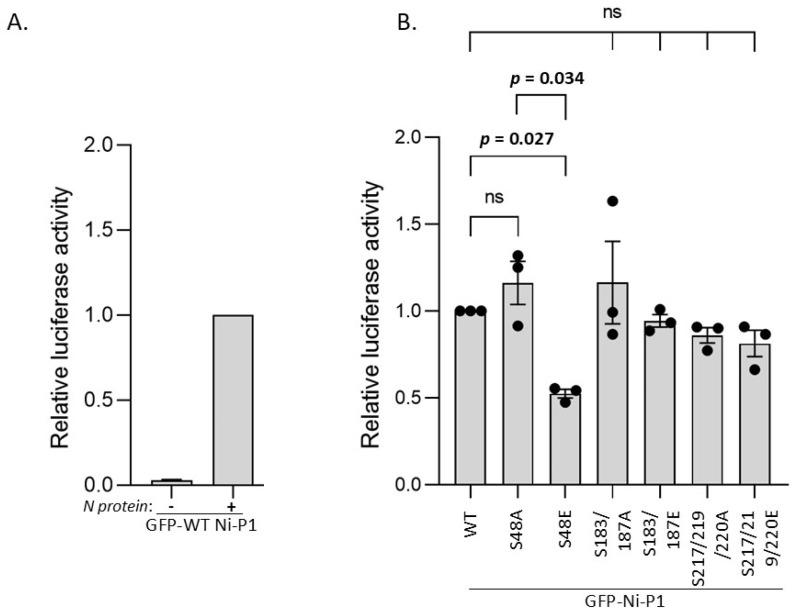
S48E impairs transcription/replication function of P1 protein. (**A**,**B**) 293T cells were transfected to express the indicated Ni P1 proteins, with RABV L protein, and with the pRVDI-luc7 minigenome construct; in (**A**), cells were cotransfected with or without plasmid to express RABV N proteins; and in (**B**), all samples we cotransfected to express N protein. Cells were lysed 48 h later for analysis of luciferase activity. Luminescence values for each sample were calculated relative to those for WT P1 (mean relative luciferase activity ± SEM, *n* = 1 assay for A (mean of 3 replicates); n = 3 assays for B, value from each assay shown as ●). Statistical analysis used Student’s *t*-test; ns, not significant.

Delivery of nascent N^0^ protein to newly synthesised viral genomic RNA requires interaction with P1 N-terminal residues 1–68 [21] (Figure 1A). Subsequent viral replication and transcription of the genome requires interaction of P with N-RNA via the P_CTD_ positive patch, and with L, dependent on P1 residues 1–19 [7,8,66] (Figure 1A). To determine whether the selected novel phosphorylation sites in the N- or C-terminal parts of P1 impact on replication/transcription function, we used a minigenome reporter assay, as previously described [24] (Figure 5). WT Ni P1 protein supported expression of luciferase (indicative of transcription/replication function) compared to a negative control sample lacking N-protein transfection (Figure 5A). P1 with the substitutions S48A, S183/S187A, S183/S187E, S217/S219/S220A, and S217/219/220E induced luciferase to levels comparable to WT P1, albeit with a minor but non-significant potential reduction for S217/S219/S220 mutants (Figure 5B). This observation is similar to those for nuclear localization (Figure 3), suggestive of a minor functional (potentially structural) effect of substitutions at S217/S219/S220; however, the lack of a defect in STAT1-antagonist function (Figure 4) suggests no major change to P_CTD_ structure. Importantly, the lack of any significant difference between the E and A substitutions of Ser183/Ser187 or of Ser217/Ser219/Ser220 indicated no specific effect of the introduction of negative charge at these positions, suggestive of no impact of phosphorylation at these sites on transcription/replication functions of P protein. However, the S48E substitution clearly reduced luciferase activity of P1 compared with WT P1 (*p* = 0.027) and S48A P1 (*p* = 0.034) (Figure 5B). This suggests that phosphorylation at Ser48 can inhibit P protein-dependent transcription/replication. As S48A P1 did not differ significantly in transcription/replication function compared with WT P1, the data further suggest that, under resting conditions, S48 of P1 is phosphorylated at relatively low levels (albeit sufficient for detection by MS), which do not substantially affect replication/transcription function. These are, to our knowledge, the first data indicating a role for phosphorylation of RABV P protein in its replication function. Phosphorylation of P proteins has been implicated in replication/transcription by mononegaviruses such as VSV (Vesicular Stomatitis Virus, family *Rhabdoviridae*, vesiculovirus genus) and measles virus (family *Paramyxoviridae*) [67,68]; our data extend phospho-regulation of P protein-dependent replication/transcription to members of the lyssavirus genus.

### 3.5. Modification at Ser48 Impacts on the Formation of Negri Body-like Structures

Ser48 is within the N^0^-binding region of P1 (residues 1–68) [21] (Figure 1A), suggesting that phosphorylation of this residue might impact on P-N^0^ interaction, potentially by increasing the negative charge on the IDR1 of P1 and so facilitating interactions in the N-protein RNA-binding groove and/or regulating the release of N^0^ for delivery to RNA. Previous structural data on the P-N^0^ interaction used CVS-11 P1 [21], which does not contain Ser48 and has an uncharged Asn48 that cannot undergo phosphorylation (although other proximal residues, for example, Ser63/64, may be phosphorylated; Figure 1 and Figure 2 [33]). The crystal structure of CVS-11 P-N^0^ showed that P protein occludes RNA interaction by binding to N-protein residues Arg149 and Arg225; molecular dynamics also predicted that N-protein residues Arg434, Arg323 and Tyr161 may contribute polar contacts [21]. Altered P-N^0^ interactions due to phosphorylation could influence the release of sequestered N protein, which would be predicted to impact on nucleocapsid formation and transcription/replication that occurs in cytoplasmic viral factories [69]. RABV replication factories (known as Negri bodies) are liquid condensates formed through interactions of P protein, N protein, and viral RNA, together with a number of host factors [42,69,70]; they can be recapitulated in a minimal system by co-expression of P- and N-proteins in cells, which results in the formation of Negri body-like structures (NBLs). This minimal system enables assessment of the impacts of mutations or truncations of P or N proteins [69,70]. Thus, if S48E impacts on transcription/replication through altered delivery of N^0^ protein to RNA, this may result in altered NBL formation by S48E P1 compared with WT P1. We would also expect wild-type and S48A to produce a similar phenotype, based on results from our minigenome analysis (see above).

**Figure 6:**
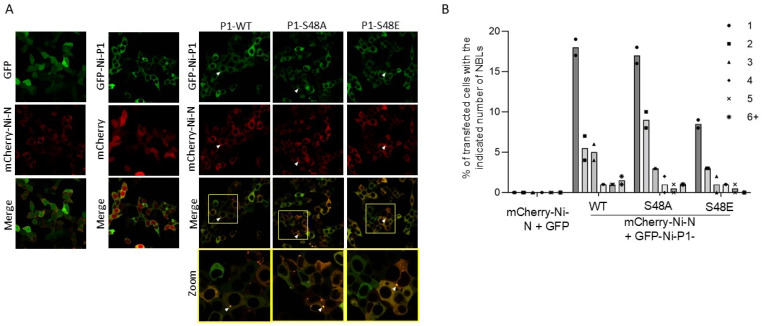
NBL formation in cells transfected to express Ni-P1 and Ni-N proteins is impaired by S48E mutation of P1. (**A**) 293T cells were transfected to express the indicated proteins and CLSM analysis of live cells was performed 24 h later. Arrowheads indicate examples of NBLs. The yellow boxes in the Merge panels are expanded in the corresponding Zoom panels (**B**) NBLs present in cells expressing both mCherry-N and GFP-P1 protein were counted, and the number of cells containing the indicated number of NBLs was calculated as a percentage of the total number of P1 and N protein co-expressing cells (data from 2 separate assays; *n* ≥ 170 cells from ≥5 fields of view for each protein).

To assess effects of S48E substitution in P1 on NBLs, we used confocal laser scanning microscopy (CLSM) to analyse living cells co-transfected to express GFP-P1 WT, S48A, or S48E with mCherry-N protein, as previously described [42,70], confirming that expression of the WT proteins results in the formation of NBLs (Figure 6A). NBLs were not apparent in control cells expressing GFP with mCherry N protein or GFP-Ni-P1 with mCherry (Figure 6A). Analysis of digitised images to assess the number of NBLs in each cell indicated that, for cells co-expressing N protein with WT P1 or P1 S48A, the proportion of the cell population in which one or more NBLs could be detected was comparable (>30% of transfected cells), as was the distribution of the number of NBLs per cell. However, the proportion of cells with detectable NBLs, and the distribution of the number of NBLs per cell were clearly impaired for cells co-expressing N protein with P1 S48E (Figure 6B), consistent with the reduced replication/transcription function of this mutant (Figure 5).

Previous data indicate that P protein regions comprising the dimerization domain, IDR2 and P_CTD_ (which are within residues 87–297) are required to form NBLs, with deletion of residues 139–151 of the IDR2 preventing NBL association [60]. Although quantitation was not reported, these data indicated that the N-terminal residues 1–86 of P protein (containing the N protein-binding site) are not critical to NBL generation such that N^0^ binding per se is not required to form NBLs. However, regulated binding of P1 to N^0^ would be expected to impact on Negri body/NBL formation where an enhanced interaction of P1-N^0^ and/or impaired release of the N^0^ protein from the P1-N^0^ complex to bind to RNA would be expected to negatively regulate formation of the P-N-RNA complexes. Such a modulatory role would be consistent with the partial but significant impact of S48E on replication (Figure 5) and NBL formation (Figure 6).

### 3.6. Expression and Crystallisation of Recombinant N^0^-P^1−52^ Complexes

Two previous crystal structures of P-N^0^ complexes have been reported: CVS-11 strain N^0^P (P1–68 with N^0^) [21] and Australian bat lyssavirus N^0^P (P1–53) [42]. CVS-11 P protein does not contain a Ser at position 48 (residue 48 is Asn), but does have phosphorylation sites at Ser63/Ser64 (Figure 1B), while ABLV P protein does contain a Ser at position 48 [42]. These Ser residues remain unresolved in the reported crystal structures; however, they are expected to be in an unphosphorylated state, consistent with the absence of post-translational modifications on proteins expressed in *E. coli*. Therefore, we expressed, purified and crystallised WT and phospho-mimetic proteins to examine the structural effects of phosphorylation at Ser48.

Expression of the N and P proteins separately in *E. coli* resulted in N protein aggregation and prevented protein purification and any subsequent biophysical analysis. To overcome this, we generated plasmids to express chimeric proteins containing residues P^1−52^-TEV-N-His, a previously successful approach for P:N complexes of measles, mumps and Ebola viruses [71,72,73]. A TEV protease site separates the proteins, enabling cleavage post-purification. The chimeric protein was expressed successfully and purified using metal-affinity resin beads, followed by size exclusion chromatography (SEC), that resulted in material that showed a single, homogenous peak by SDS-PAGE.

### 3.7. Crystal Structures of Ni-CE N^0^-P and Ni N^0^-P-S48E

The purified chimeric protein yielded orthorhombic *P2_1_2_1_2_1_* symmetry crystals of both Ni-CE N^0^P and Ni N^0^P-S48E, from which X-ray diffraction data were collected at the Australian Synchrotron on the MX2 beamline (Table 1). These data were used to generate a 2 Å resolution atomic structure of the Ni-CE N^0^P complex and a 2.15 Å resolution structure of the Ni N^0^P-S48E complex. The final models had good stereochemistry and a R_work_ and R_free_ of 18.22% and 21.7%, respectively (Ni-CE N^0^P) and 18.28% and 21.48%, respectively (Ni N^0^P-S48E). Complete data collection and refinement statistics for the resolved structures are presented in Table 1. The crystal structure for Ni N^0^P was unable to be resolved.

In both crystals, the unit cell contained a single chimeric protein chain that clearly represented the N- and P-protein components, together with 386 and 316 water molecules for Ni-CE N^0^P and Ni N^0^P-S48E, respectively. The N-protein components of the structures were similar (RMSD 0.201 Å) and could be traced from residues Glu27 to Asp449 for Ni-CE N^0^P, and Tyr26 to Ser448 for Ni N^0^P-S48E. Chain breaks occurred in the N proteins (Ni-CE N^0^P residues 351–399 and Ni N^0^P-S48E residues 352–399) due to a flexible region that was not captured in the crystal structure. The tertiary structure of the N-protein chain was essentially as described previously [21,73], with N- and C-terminal domains comprising multiple helices and turns, together with a central hinge region, creating a jaw-like structure.

Good electron density was observed for the P-protein chain in both complexes and was traced from Ile4 to Gln40 in Ni-CE N^0^P. The interface was analysed using PDBsum [54] and covers 1760 Å^2^ and was mediated by two salt bridges, 13 hydrogen bonds, and 157 non-bonded contacts (Supplementary Table S1). In addition to the hydrophobic interactions, the interface was mediated by interactions with basic N-protein residues, consistent with the recent RABV CVS-11 and ABLV N^0^P structures 8B8V and 8FWL [21,42].

**Figure 7:**
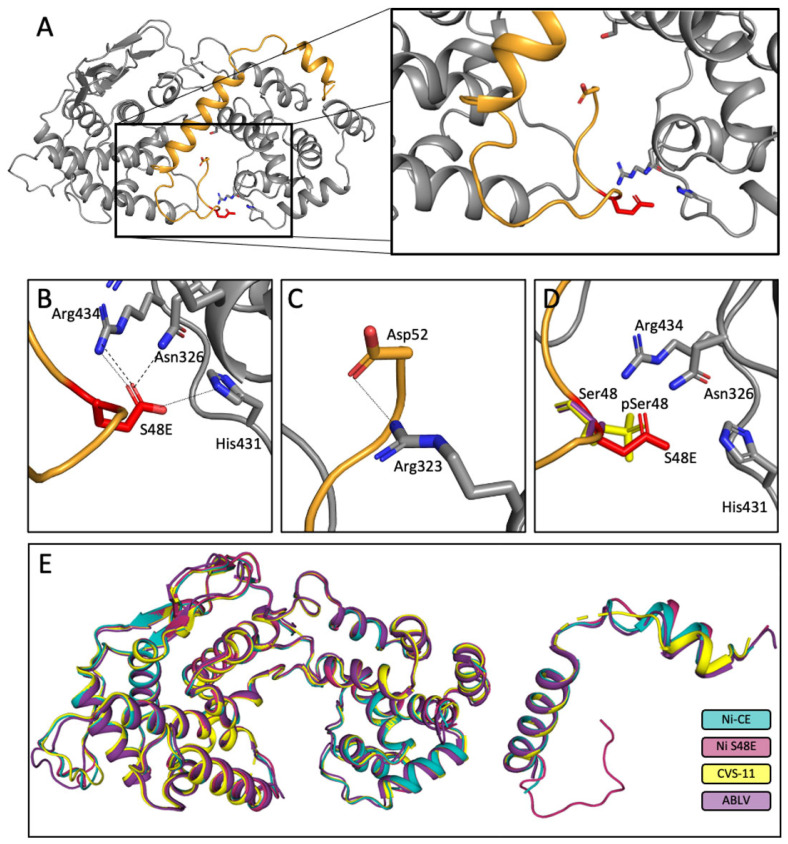
The S48E mutation enhances the interaction of P protein with N^0^. (**A**) The crystal structure of the RABV N protein (grey cartoon) and P protein interface (orange cartoon) shows an increased binding interface through stabilisation of a flexible P loop located within the N-RNA-binding groove. The inset shows the flexible loop of the P protein S48E mutant interfacing with the N protein in the RNA binding groove. (**B**) The P protein flexible loop is stabilised by salt bridges (small-dashed lines) and H-bonds (large-dashed lines) between the S48E Glu (red sticks) and N protein Asn326, His431, and Arg323 (grey sticks). (**C**) A salt bridge is also formed between P protein Asp52 and N protein Arg323. (**D**) The Glu48 (the mutation mimicking phosphorylation of Ser48) is shown as red sticks and is superimposed over a theoretical phosphorylated Ser48 (yellow sticks) and non-phosphorylated Ser48 (purple sticks). (**E**) Superimposition of the resolved lyssavirus N^0^P proteins (N protein left, P protein right), showing the additional P fragment captured in the Ni S48E crystal structure. Ni S48E is dark magenta (this study), Ni-CE (this study) is teal, CVS-11 (PDB 8B8V) is yellow, and ABLV (PDB 8FWL) is purple. Figures generated using Pymol.

In the Ni N^0^P-S48E structure, the P-protein chain could be traced from Lys3 to Asp52 with strong electron density from residues 3–40, and weaker density from Gly41-Asp52. The P-protein chain had essentially the same architecture as Ni-CE N^0^P from the N-terminus to Gln40, with differences between Ni-CE and Ni only in the orientation of the sidechains from Ile11 to Ala16. Notably, an additional 13 P-protein residues were observed in the Ni N^0^P-S48E structure, forming a somewhat flexible loop that extended through a groove that is formed between the two domains of the N protein and to which viral RNA binds (Figure 7A) [74]. The interface was analysed using PDBsum [54] and covered 2260 Å^2^ and was mediated by four salt bridges, 19 hydrogen bonds, and 221 non-bonded contacts. An additional eight hydrogen bonds were present in the Ni N^0^P-S48E structure; four between helix 2 (which comprises residues 20–39) of P protein and the N protein, and four hydrogen bonds and three salt-bridges mediated by the flexible loop of P protein formed by residues 41–52 (Supplementary Table S2). Importantly, Glu48 (the phospho-mimetic mutation for Ser48) and Asp52 of P protein formed salt bridges with Arg434 and Arg432 of N protein, respectively, which are also critical residues in the N-RNA interface [74] and Glu48 also formed a salt bridge with His490 of N protein (Figure 7A,B). The additional salt bridges formed by Glu48 suggest that the phosphorylation of Ser48, producing an analogous interaction, may enhance the stability of the N^0^P interaction and potentially impair the release of P from N^0^P complexes and subsequent binding/encapsidation of viral RNA could be affected, thus inhibiting replication/transcription. 

Together, these data indicate that the S48E mutation enhances the interaction of P1 with N^0^, which is likely to result in reduced NBL formation in cells (Figure 6), probably via enhanced occlusion of the RNA binding region/reduced delivery of the N protein to RNA, thus reducing transcription/replication function (Figure 5). We propose that the identity of the phosphorylated motif is strain-dependent, and that function is driven by charge complementarity, rather than strict sequence specificity. Furthermore, the inherent flexibility and length of P protein’s IDR1 region may facilitate dynamic positioning of different phosphorylation sites within the RNA-binding groove. Further investigations are needed to evaluate this. The kinases associated with phosphorylation of the P protein N-terminus are poorly defined, but a recent report suggested that kinases of the IFN induction pathway may phosphorylate CVS Ser63/Ser64 [34]. This could potentially facilitate a mechanism by which the virus can monitor and respond to the IFN induction pathway, resulting in replication being suppressed during an active immune response and then reactivated following viral inhibition of the response, an alternative function of P protein [4]. Such a mechanism is consistent with the observation that S48A P1 behaves similarly to WT P1 in the minigenome assay (Figure 5), indicating that S48 is minimally phosphorylated in resting cells, requiring specific stimulation to enhance phosphorylation, to suppress replication The idea that phosphorylation might control a switch between replication and immune evasion, both of which employ mechanisms involving P protein, is an attractive hypothesis because phosphorylation is a reversible post-translation modification, and so would facilitate dynamic switching between these processes, as required.

Overall, while the initial findings are compelling and suggest a plausible mechanism by which phosphorylation may influence viral replication processes, a limitation of this study is that dissociation constant data were not collected. Future research should include quantification of the protein interactions which should further clarify the intricate interplay between phosphorylation events and viral protein dynamics.

In summary, our analysis has identified a number of novel sites/regions of RABV P protein that are phosphorylated in cells, and characterised functionally three of these. The characterised sites/regions did not appear to have any substantial impact on nuclear trafficking or IFN-antagonist functions of P1. Phosphorylation at Ser48, however, appeared to significantly reduce the replication function of P1. We postulate that this is due to an increased affinity of P protein at residues 41–52 for the N protein via facilitated binding in the N protein RNA-binding groove, which in turn reduces the formation of NBLs.

## Figures and Tables

**Table 1:** Data collection and refinement statistics for Ni-CE N^0^-P and Ni N^0^P-S48E.

Data Collection and Processing	Ni-CE N^0^P	Ni N^0^P-S48E
Wavelength (Å)	0.9537	0.9537
Resolution range (Å)	29.90–2.00 (2.05–2.00)	29.88–2.10 (2.16–2.10)
Space group	P 21 21 21	P 21 21 21
Unit cell (Å, °)	42.17 74.50 154.72, 90 90 90	41.89 75.39 154.99, 90 90 90
Total reflections	388,562 (22,768)	138,385 (10,791)
Unique reflections	33,217 (2309)	29,538 (2335)
Multiplicity	11.7 (9.9)	4.7 (4.6)
Completeness (%)	98.1 (95.1)	99.9 (99.8)
Mean I/sigma(I)	12.4 (1.8)	5.9 (2.0)
Wilson B-factor Å^2^	23.177	21.811
R-merge %	0.141 (1.380)	0.138 (0.854)
R-pim %	0.042 (0.428)	0.071 (0.456)
CC1/2	0.997 (0.656)	0.989 (0.849)
**Refinement**		
Resolution range (Å)	20.0–2.0	20.0–2.15
Number of reflections	33,008	27,965
Number of R-free reflections	1718	1474
R-work %	18.22	18.28
R-free	21.70	21.48
RMS (bonds)	0.003	0.003
RMS (angles)	0.60	0.58
Ramachandran plot		
favoured (%)	98.26	98.10
allowed (%)	1.74	1.90
outliers (%)	0	0
**Validation**		
Clash score	0.62	2.22
MolProbity score (percentile)	0.71 (100)	1.00 (100)
**PDB accession code**	8U0A	8U0B

Statistics for the highest-resolution shell are shown in parentheses.

## Data Availability

Dataset available on request from the authors.

## Supplementary Materials

The following supporting information can be downloaded at: https://www.mdpi.com/article/10.3390/v17081075/s1, Figure S1. Expression of P proteins and mutants. (A,B) 293T cells were transfected to express the indicated proteins for 24 h before (A) analysis of living cells using fluorescence microscopy (scale bar, 400 mm) or (B) lysis and analysis by immunoblotting (IB) using anti-GFP antibody; Figure S2: Byonic software output showing peptide coverage and post translation modifications of Ni P1 protein. Spectral data of the peptides that identified phosphorylation within Ni P1 protein. Blue indicate B ions and red indicate Y ions. Spectra are from a single assay (3 replicate assays were performed for MS analysis); Table S1. Interacting residues at the interface of RABV NiCE-N^0^P as determined by PDBsum; Table S2. Interacting residues at the interface of RABV Ni-N^0^P-S48E as determined by PDBsum.
