# Supplementary material for: The Replication Function of Rabies Virus P Protein Is Regulated by a Novel Phosphorylation Site in the N-Terminal N Protein-Binding Region
Source: Viruses. 2025 Aug 1;17(8):1075. doi: 10.3390/v17081075 (PMC12390708; doi:10.3390/v17081075)
Supplement: Supplementary file 1 [file viruses-17-01075-s001.zip › viruses-3699080-supplementary.pdf]

Replication function of rabies virus P protein is regulated by a novel phosphorylation site in the N-terminal N protein-binding region.

Supplementary Figures and Tables

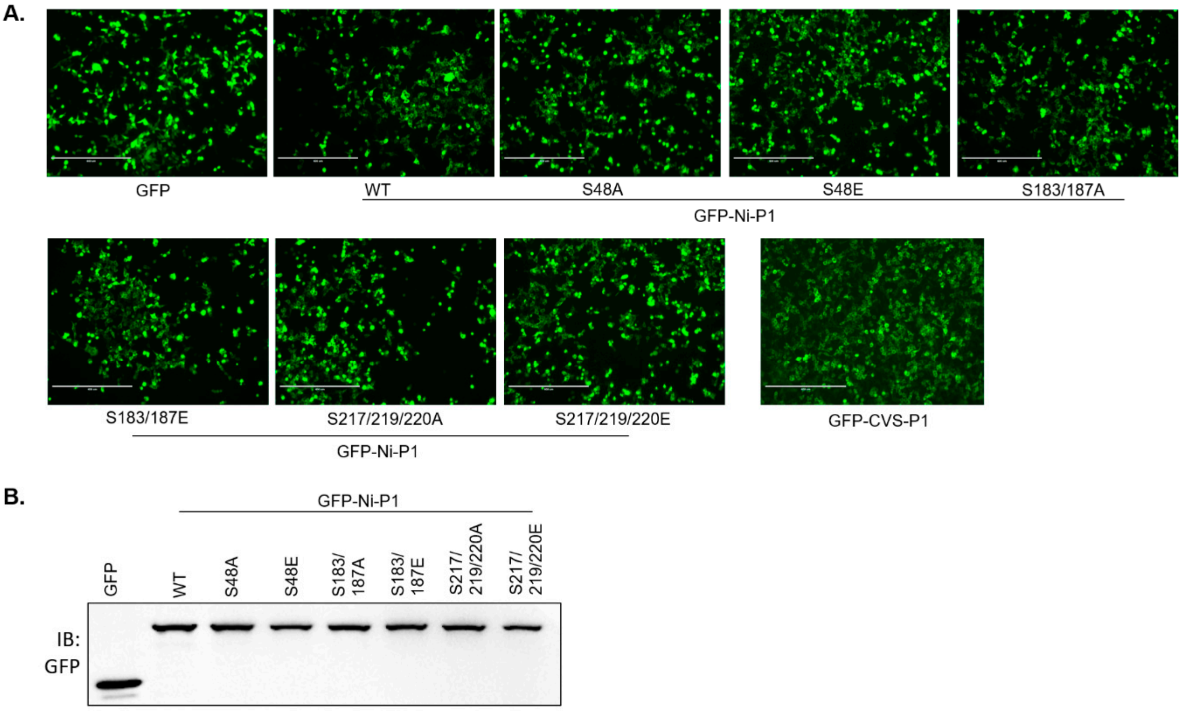

Supplementary Figure S1. Expression of P proteins and mutants. (A,B) 293T cells were transfected to express the indicated proteins for 24 h before (A) analysis of living cells using fluorescence microscopy (scale bar: 400mm), or (B) lysis and analysis by immunoblotting (IB) using anti-GFP antibody.

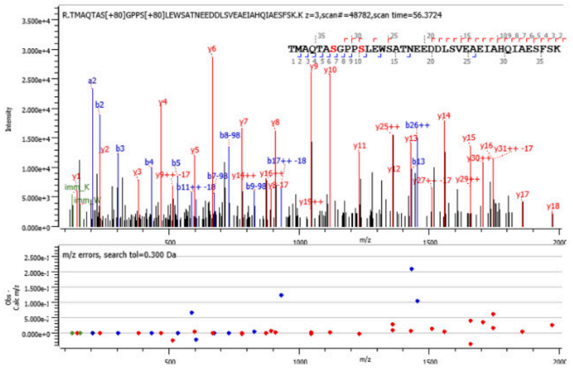
R.TMAQTAS[+80]GPPS[+80]LEWSATNEEDDLSVEAEIAHQIAESFSK.K z=3,scan#=48782,scan time=56.3724
m/z errors, search tol=0.300 Da

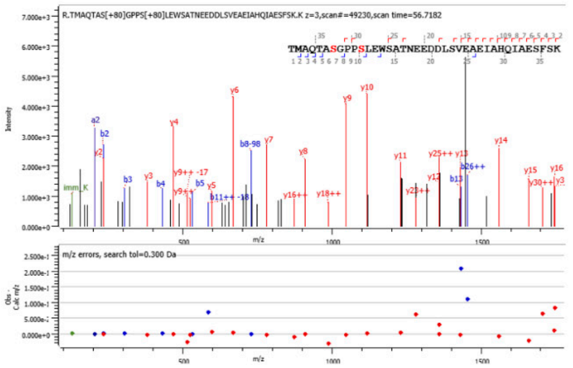
R.TMAQTAS[+80]GPPS[+80]LEWSATNEEDDLSVEAEIAHQIAESFSK.K z=3,scan#=49230,scan time=56.7182
m/z errors, search tol=0.300 Da

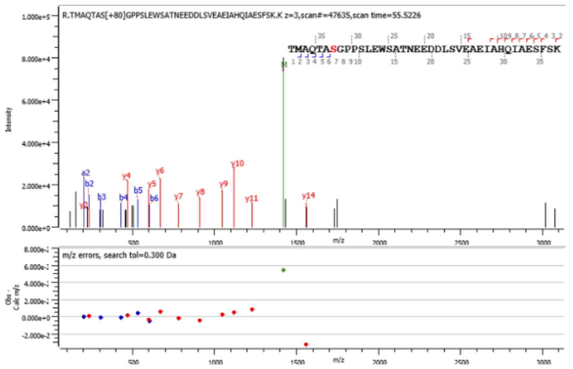
R.TMAQTAS[+80]GPPSLEWSATNEEDDLSVEAEIAHQIAESFSK.K z=3,scan#=47635,scan time=55.5226
m/z errors, search tol=0.300 Da

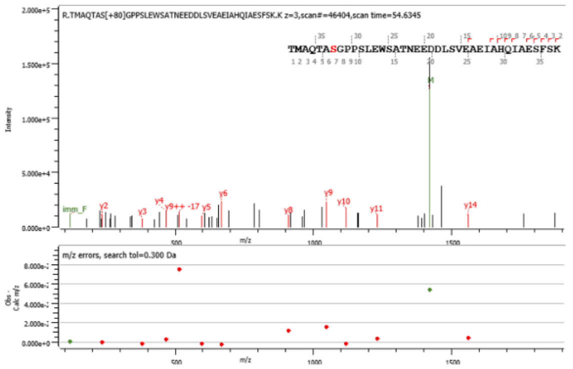
R.TMAQTAS[+80]GPPSLEWSATNEEDDLSVEAEIAHQIAESFSK.K z=3,scan#=46404,scan time=54.6345
m/z errors, search tol=0.300 Da

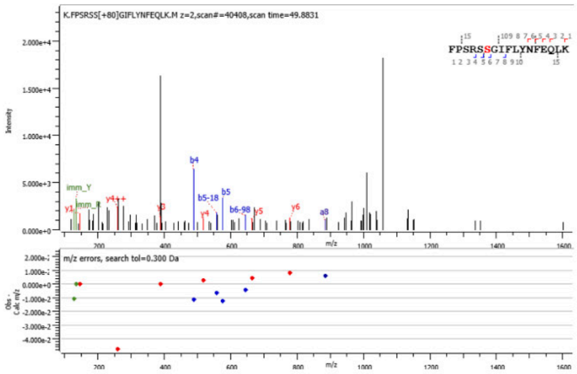
K.FPSRSS[+80]GIFLYNFEQLK.M z=2,scan#=40408,scan time=49.8831
m/z errors, search tol=0.300 Da

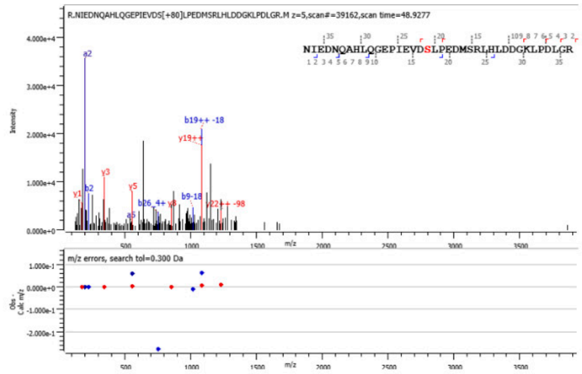
R.NIEDNQAHLQGEPIEVDS[+80]LPEDMSRLHLDDGKLPDLGR.M z=5,scan#=39162,scan time=48.9277
m/z errors, search tol=0.300 Da

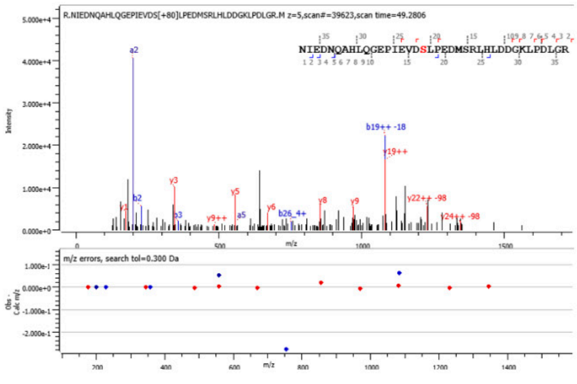
R.NIEDNQAHLQGEPIEVDS[+80]LPEDMSRLHLDDGKLPDLGR.M z=5,scan#=39623,scan time=49.2806
m/z errors, search tol=0.300 Da

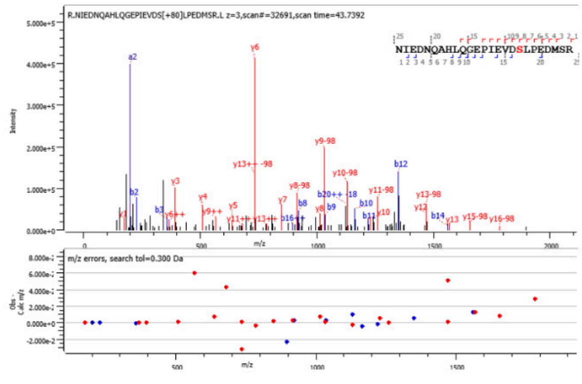
R.NIEDNQAHLQGEPIEVDS[+80]LPEDMSR.L z=3,scan#=32691,scan time=43.7392
m/z errors, search tol=0.300 Da

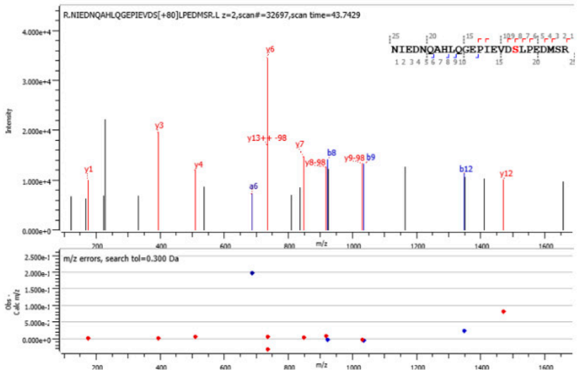
R.NIEDNQAHLQGEPIEVDS[+80]LPEDMSR.L z=2,scan#=32697,scan time=43.7429
NIEDNQAHLQGEPIEVDSLPEDMSR
m/z errors, search tol=0.300 Da

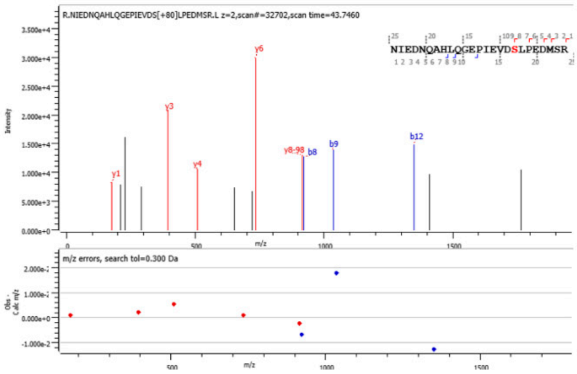
R.NIEDNQAHLQGEPIEVDS[+80]LPEDMSR.L z=2,scan#=32702,scan time=43.7460
NIEDNQAHLQGEPIEVDSLPEDMSR
m/z errors, search tol=0.300 Da

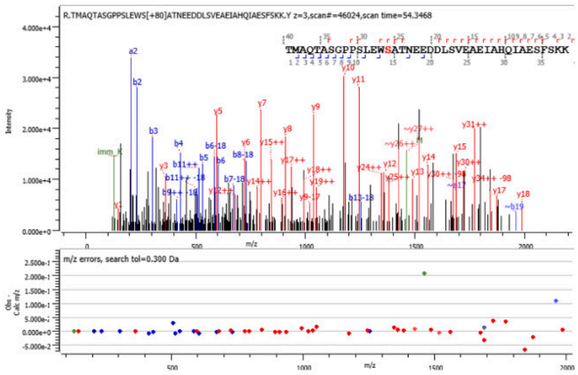
R.TMAQTASGPPSLEWS[+80]ATNEEDDLSVEAEIAHQIAESFSKK.Y z=3,scan#=46024,scan time=54.3468
TMAQTASGPPSLEWSATNEEDDLSVEAEIAHQIAESFSKK
m/z errors, search tol=0.300 Da

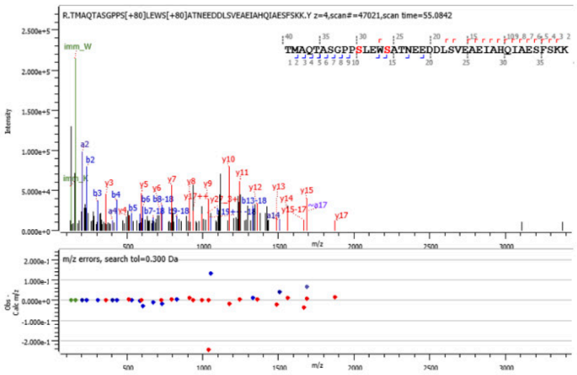
R.TMAQTASGPPS[+80]LEWS[+80]ATNEEDDLSVEAEIAHQIAESFSKK.Y z=4,scan#=47021,scan time=55.0842
TMAQTASGPPSLEWSATNEEDDLSVEAEIAHQIAESFSKK
m/z errors, search tol=0.300 Da

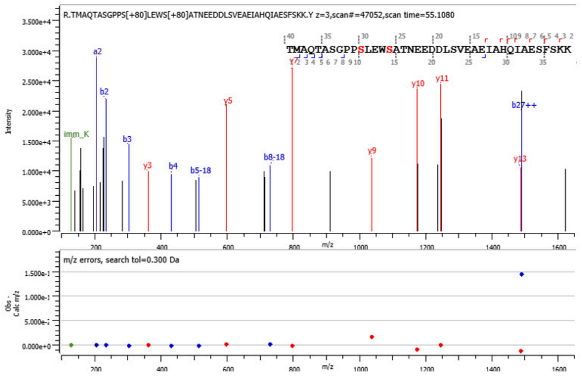
R.TMAQTASGPPS[+80]LEWS[+80]ATNEEDDLSVEAEIAHQIAESFSKK.Y z=3,scan#=47052,scan time=55.1080
TMAQTASGPPSLEWSATNEEDDLSVEAEIAHQIAESFSKK
m/z errors, search tol=0.300 Da

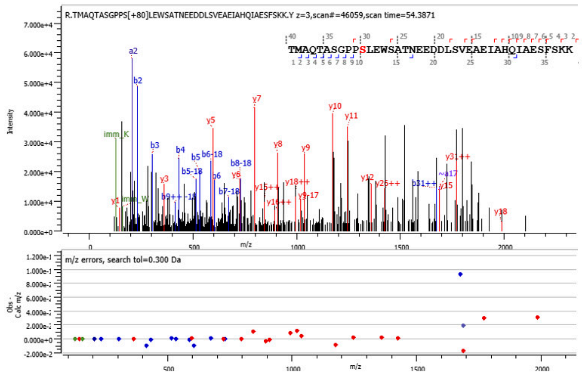
R.TMAQTASGPPS[+80]LEWSATNEEDDLSVEAEIAHQIAESFSKK.Y z=3,scan#=46059,scan time=54.3871
TMAQTASGPPSLEWSATNEEDDLSVEAEIAHQIAESFSKK
m/z errors, search tol=0.300 Da

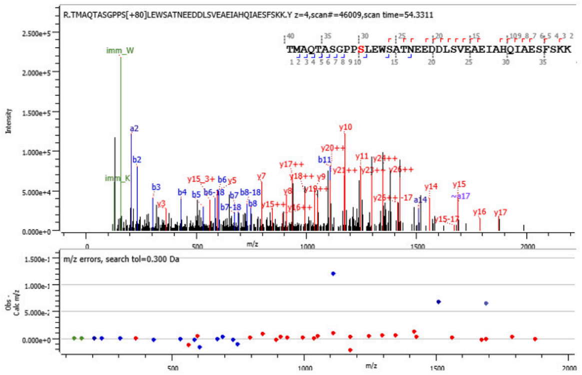
R.TMAQTASGPPS[+80]LEWSATNEEDDLSVEAEIAHQIAESFSKK.Y z=4,scan#=46009,scan time=54.3311
TMAQTASGPPSLEWSATNEEDDLSVEAEIAHQIAESFSKK
m/z errors, search tol=0.300 Da

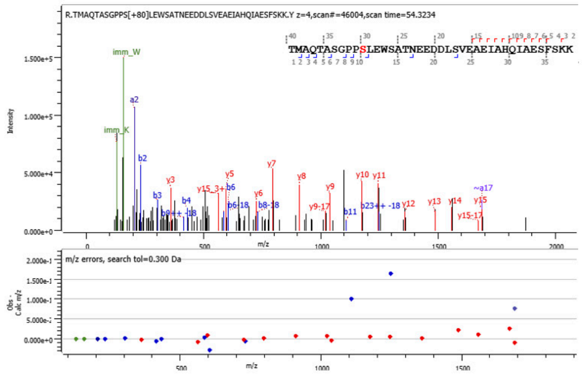
R.TMAQTASGPPS[+80]LEWSATNEEDDLSVEAEIAHQIAESFSKK.Y z=4,scan#=46004,scan time=54.3234
TMAQTASGPPSLEWSATNEEDDLSVEAEIAHQIAESFSKK
m/z errors, search tol=0.300 Da

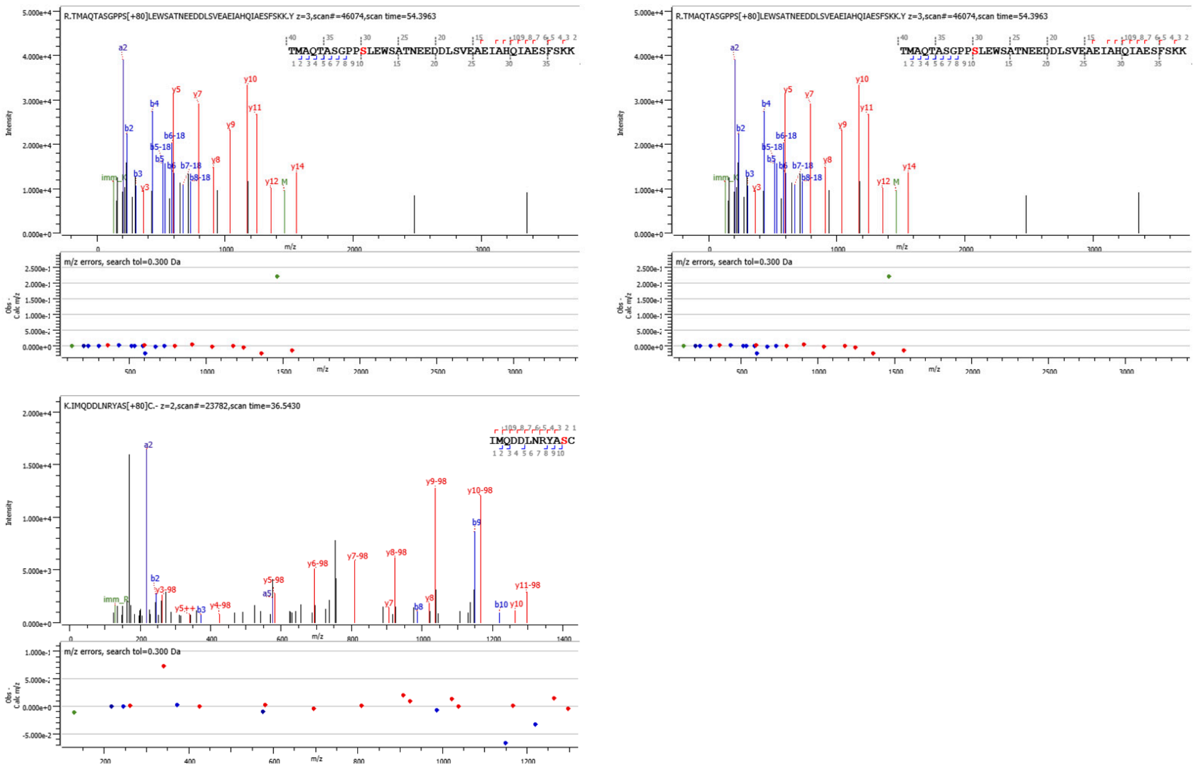

Supplemental Figure S2: Byonic software output showing peptide coverage and post translation modifications of Ni P1 protein. Spectral data of the peptides that identified phosphorylation within Ni P1 protein. Blue indicates B ions and red indicates Y ions. Spectra are from a single sample from one assay (3 replicate assays were performed).

Nucleoprotein					Phosphoprotein		
Hydrogen bonds							
1	261	Phe	O	<-->	6	Val	N
2	270	Arg	NH1	<-->	17	Asp	OD2
3	253	Ala	N	<-->	22	Glu	OE1
4	254	Arg	N	<-->	22	Glu	OE2
5	279	Thr	OG1	<-->	24	Thr	OG1
6	243	Thr	O	<-->	29	Asn	ND2
7	243	Thr	OG1	<-->	29	Asn	OD1
8	233	Tyr	OH	<-->	31	Asn	ND2
9	225	Arg	NH2	<-->	35	Asn	OD1
10	168	Arg	NH2	<-->	37	Ala	O
11	168	Arg	NH1	<-->	38	His	O
12	149	Arg	NH1	<-->	39	Leu	O
13	149	Arg	NH2	<-->	39	Leu	O
Salt bridges							
1	403	Glu	OE1	<-->	12	Arg	NH2
2	270	Arg	NH1	<-->	17	Asp	OD2
Non-bonded contacts							
157							

Supplementary Table S1. Interacting residues at the interface of RABV NiCE-N^{0}P as determined by PDBsum

Nucleoprotein					Phosphoprotein		
Hydrogen bonds							
1	263	Lys	N	<-->	4	Ile	O
2	261	Phe	O	<-->	6	Val	N
3	270	Arg	NH2	<-->	14	Gly	O
4	254	Arg	NH1	<-->	15	Leu	O
5	254	Arg	NH2	<-->	16	Ala	O
6	254	Arg	N	<-->	22	Glu	OE1
7	279	Thr	OG1	<-->	24	Thr	OG1
8	279	Thr	OG1	<-->	24	Thr	OG1
9	243	Thr	O	<-->	29	Asn	ND2
10	243	Thr	OG1	<-->	29	Asn	OD1
11	233	Tyr	OH	<-->	31	Asn	ND2
12	225	Arg	NH2	<-->	35	Asn	OD1
13	168	Arg	NH2	<-->	37	Ala	O
14	168	Arg	NH1	<-->	38	His	O
15	149	Arg	NH1	<-->	38	His	O
16	149	Arg	NH2	<-->	39	Leu	O
17	326	Asn	ND2	<-->	48	Glu	OE1
18	434	Arg	NH2	<-->	48	Glu	OE1
19	237	Arg	OG	<-->	52	Asp	O
Salt bridges							
1	403	Glu	OE2	<-->	12	Arg	NH2
2	434	Arg	NH2	<-->	48	Glu	OE1
3	431	His	NE2	<-->	48	Glu	OE2
4	323	Arg	NH2	<-->	52	Asp	OD1
Non-bonded contacts							
212							

Supplementary Table S2. Interacting residues at the interface of RABV Ni-N^{0}P-S48E as determined by PDBsum
